# Supplementary material for: Regulatory Role of Sphingosine-1-Phosphate and C16:0 Ceramide, in Immunogenic Cell Death of Colon Cancer Cells Induced by Bak/Bax-Activation
Source: Cancers (Basel). 2022 Oct 22;14(21):5182. doi: 10.3390/cancers14215182 (PMC9657779; doi:10.3390/cancers14215182)
Supplement: Supplementary file 1 [file cancers-14-05182-s001.zip › cancers-1974772-supplementary.pdf]

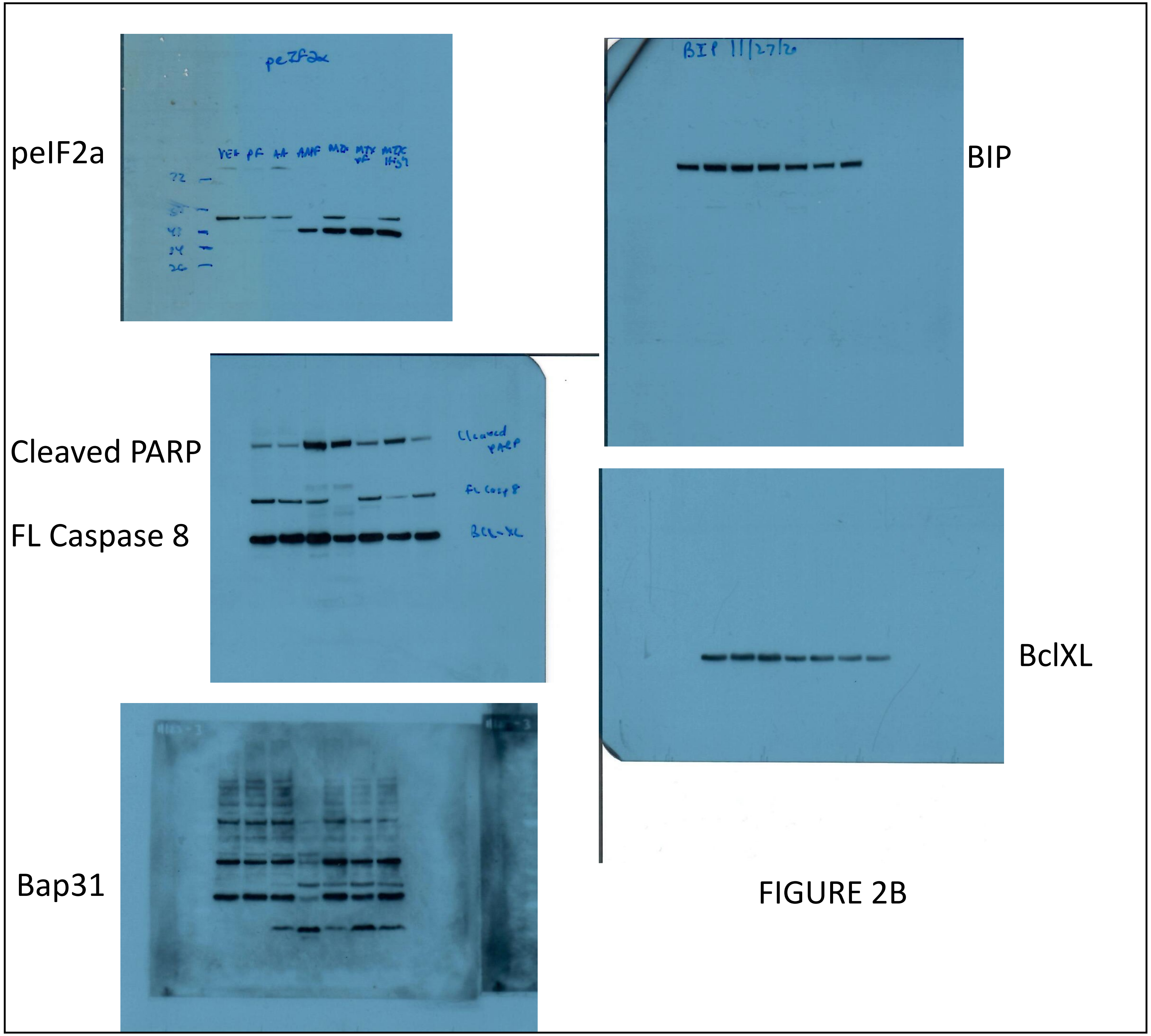

FIGURE 2B

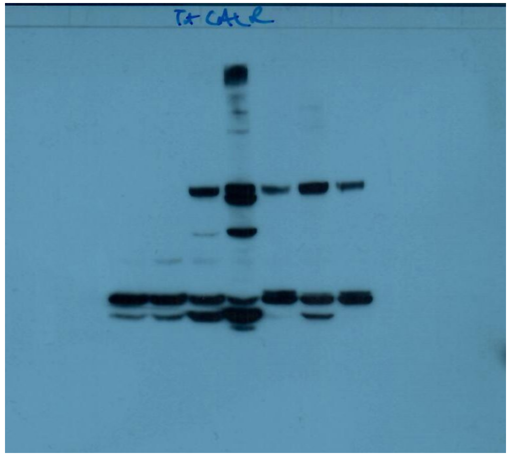
Figure 2C
CALR

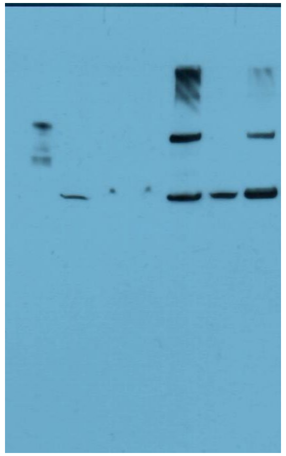
Figure 2E
CALR

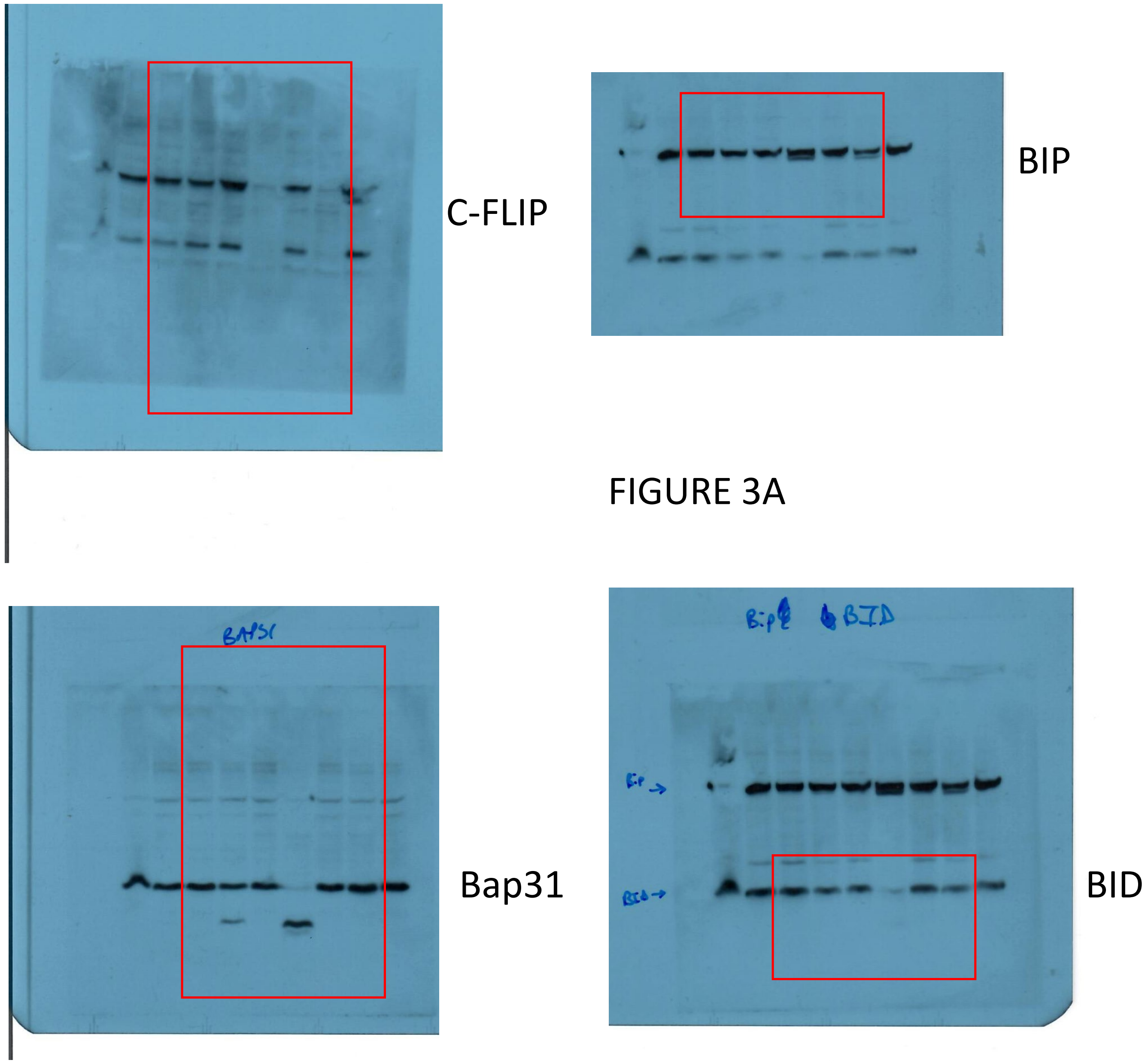

FIGURE 3A

FIGURE 3B

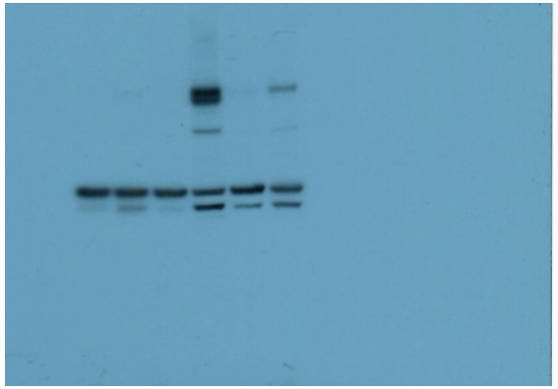

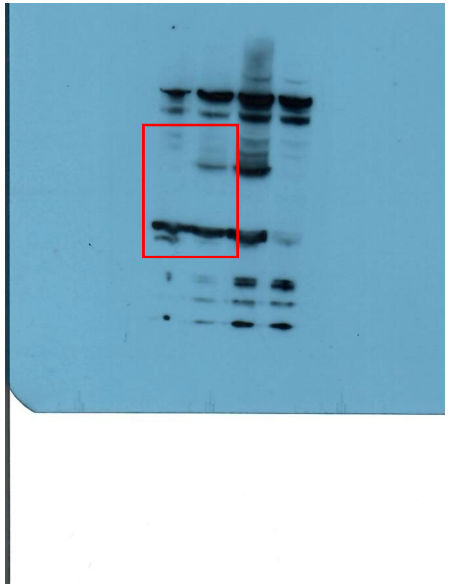

FIGURE 4A
GFP and
endo SphK1

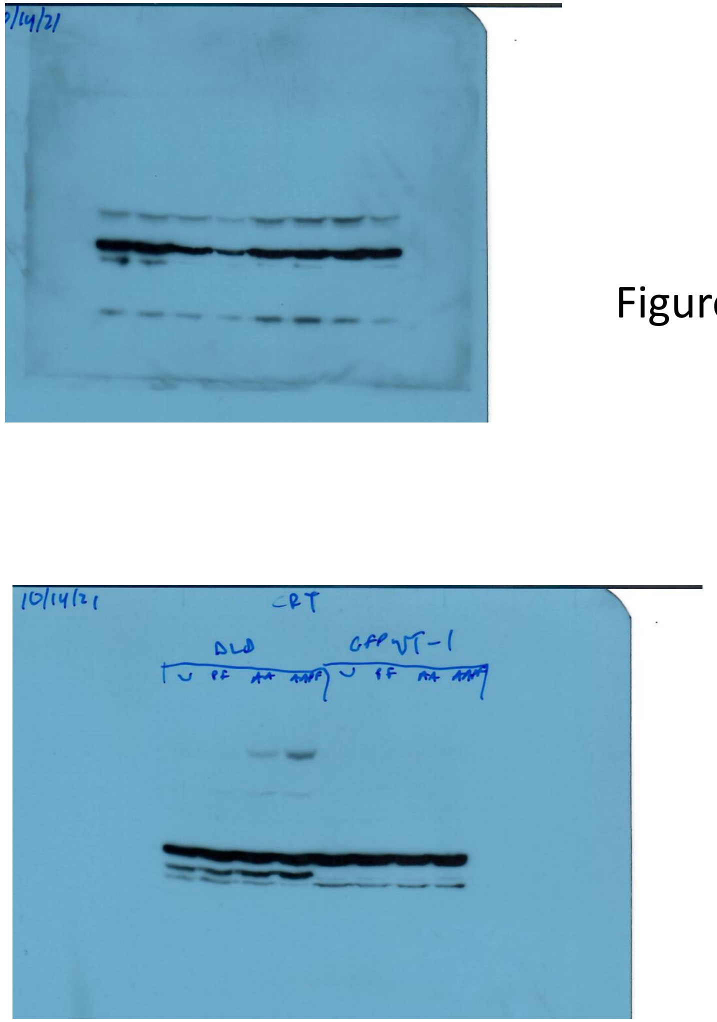

Figure 4D

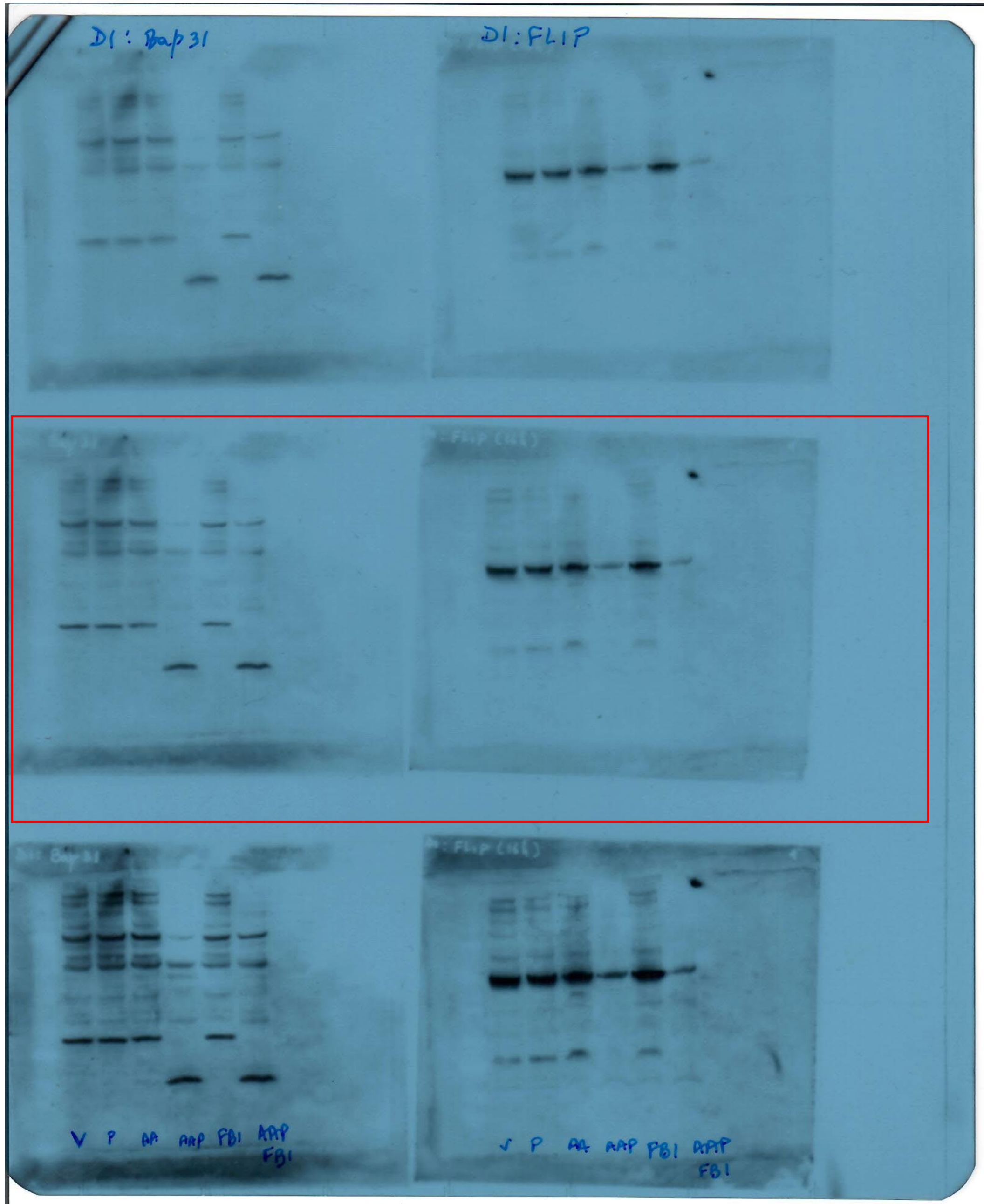

FIGURE 5A BAP31 and C-FLIP

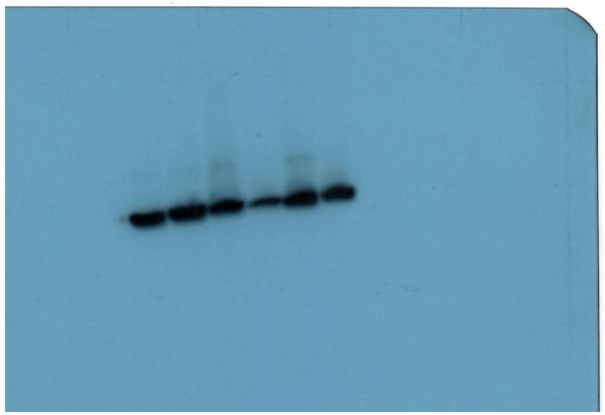

FIGURE 5A
GAPDH

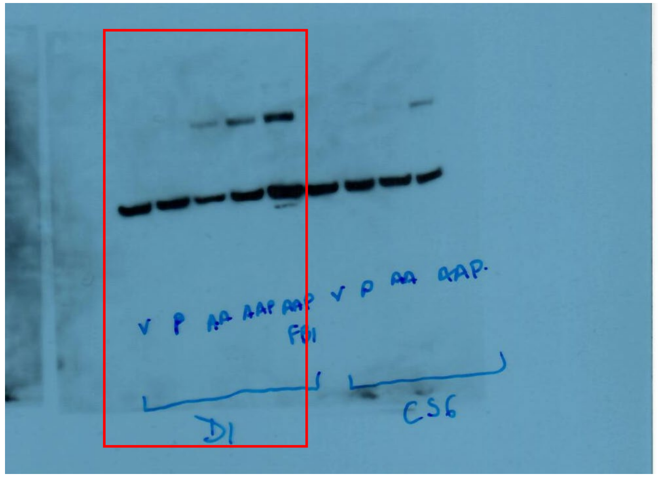

FIGURE 5B
CALR

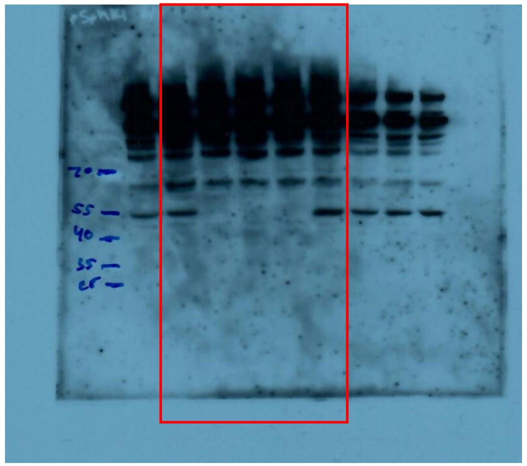

Figure 6A
phosphoSphK1

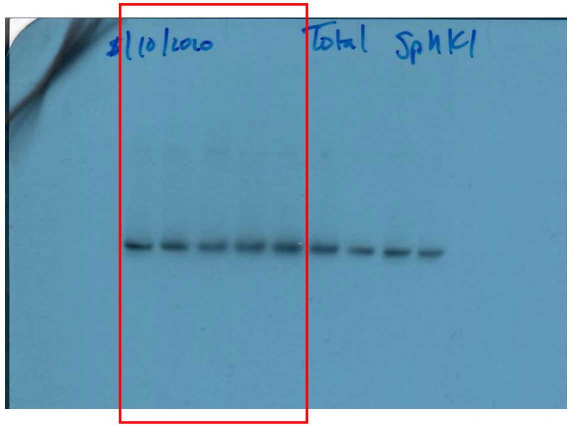

Figure 6B
TotalSphK1

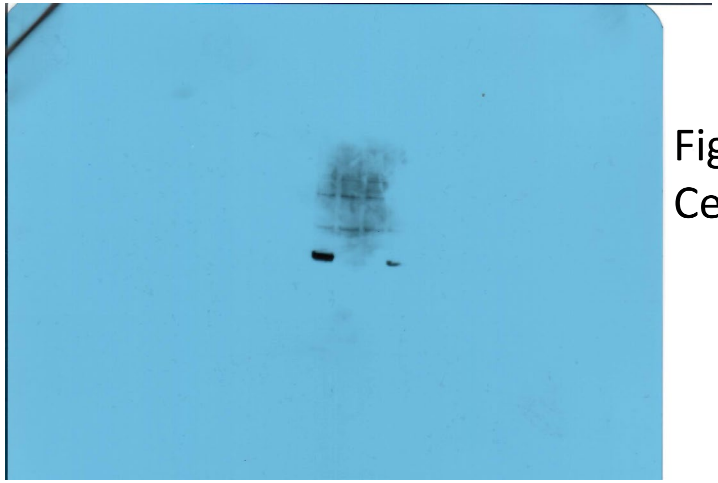

Figure 8A
Cers6

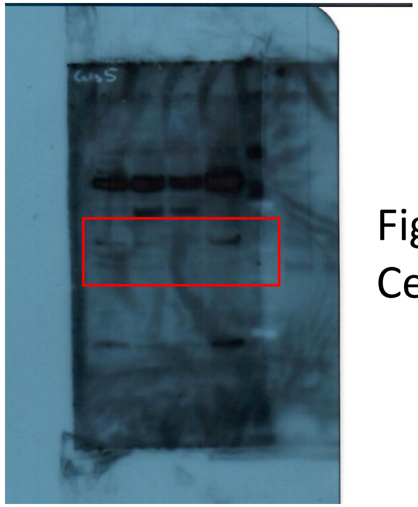

Figure 8C
Cers5

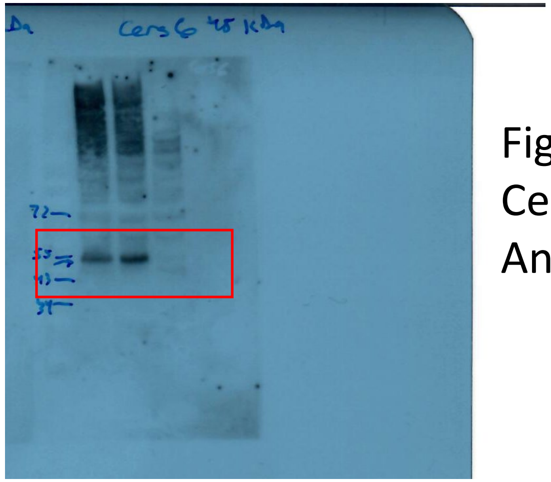

Figure 8E
Cers6
And GAPDH

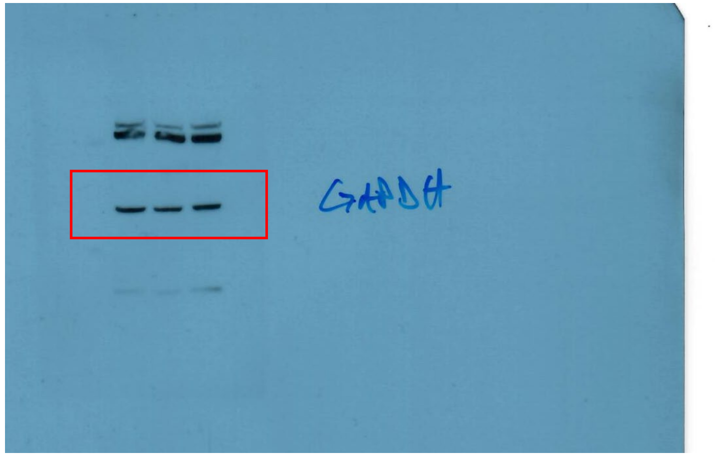

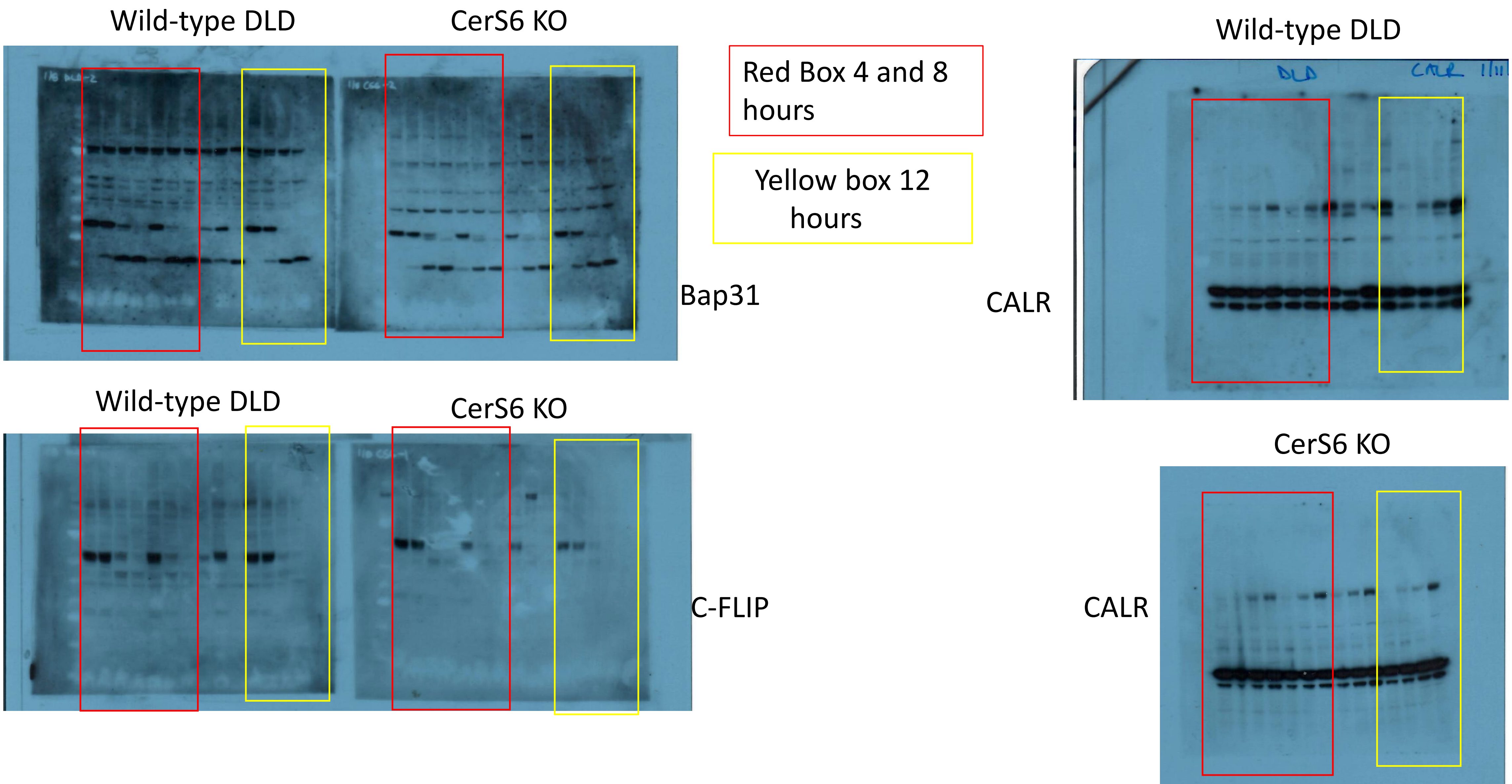
Wild-type DLD
CerS6 KO
Red Box 4 and 8 hours
Yellow box 12 hours
Bap31
Wild-type DLD
CerS6 KO
C-FLIP
Wild-type DLD
CALR
CerS6 KO
CALR

FIGURE 8F
